# Chemometric Classification of Colombian Cacao Crops: Effects of Different Genotypes and Origins in Different Years of Harvest on Levels of Flavonoid and Methylxanthine Metabolites in Raw Cacao Beans

**DOI:** 10.3390/molecules27072068

**Published:** 2022-03-23

**Authors:** Catalina Agudelo, Susana Acevedo, Luis Carrillo-Hormaza, Elkin Galeano, Edison Osorio

**Affiliations:** 1 Grupo de Investigación en Sustancias Bioactivas, Facultad de Ciencias Farmacéuticas y Alimentarias, Universidad de Antioquia, Calle 70 No. 52-21, Medellín 0500100, Colombia; catalina.agudelor@udea.edu.co (C.A.); susana.munoza@udea.edu.co (S.A.); lcarrillo@bioingred.co (L.C.-H.); elkin.galeano@udea.edu.co (E.G.); 2 Bioingred, Spin-Off Universidad de Antioquia, Itagüí 055412, Colombia

**Keywords:** *Theobroma cacao*, cocoa clones, catechins, methylxanthines, procyanidins

## Abstract

The aim of this study was to evaluate the levels of chemical markers in raw cacao beans in two clones (introduced and regional) in Colombia over several years. Multivariate statistical methods were used to analyze the flavanol monomers (epicatechin and catechin), flavanol oligomers (procyanidins) and methylxanthine alkaloids (caffeine and theobromine) of cocoa samples. The results identified genotype as the main factor contributing to cacao chemistry, although significant differences were not observed between universal and regional clones in PCA. The univariate analysis allowed us to establish that EET-96 had the highest contents of both flavanol monomers (13.12 ± 2.30 mg/g) and procyanidins (7.56 ± 4.59 mg/g). In addition, the geographic origin, the harvest conditions of each region and the year of harvest may contribute to major discrepancies between results. Turbo cocoa samples are notable for their higher flavanol monomer content, Chigorodó cocoa samples for the presence of both types of polyphenol (monomer and procyanidin contents) and the Northeast cocoa samples for the higher methylxanthine content. We hope that knowledge of the heterogeneity of the metabolites of interest in each clone will contribute to the generation of added value in the cocoa production chain and its sustainability.

## 1. Introduction

Cocoa (*Theobroma cacao L*.) is a plant of Latin American origin that is widely cultivated in many tropical regions around the world. Cocoa is the essential ingredient in chocolate and other cocoa products, and 4.84 million tons of cocoa beans were produced worldwide during 2020 [1]. Additionally, in 2019, the cocoa product market earned $24.5 billion, and it is projected to have an estimated value of $30.2 billion in 2026, as the global demand is stronger than ever [2]. As a result, cocoa crops have seen rapid increases in the variety of cocoa clones and the development of regional varieties generated from controlled pollination or hybridization in producing countries [3]. In Colombia, the production of cocoa beans is a strategic business sector, and the country is recognized by the International Cocoa Organization as being among a select group of countries that are producers of “Fine Cocoa” [4]. Generally, different cocoa clones exist in three core groups: Criollo, Forastero and Trinitario. The Colombian cacao germplasm hosts introduced clones (universal clones) and regional varieties [5]. Cacao regional clones with higher yields have been developed to obtain production levels that enable growers to meet demand without sacrificing tolerance to plague and diseases [6]. The Nacional Federation of Cacao (Fedecacao) in Colombia has conducted studies with more than 100 promising clones. For example, Federación Arauquita (FEAR-5), Federación El Carmen (FEC-2), Federación San Vicente (FSV-41) and Federación Tame 2 (FTA-2) are clones with high agricultural penetration. The commercial/universal clones Castro Naranjal Collection (CCN51) and Imperial College Selection (ICS-95) have also been cultivated in Colombia. These clones are disease-resistant and are known for their high yields [7,8].

Each cacao clone provides beans with a characteristic chemical composition and sensorial profile related to its origin [9,10]. However, the quality of the cocoa beans also depends on environmental conditions (weather and soil properties), the age of the cocoa tree and the different biochemical reactions occurring in the beans during the postharvest techniques [11,12,13]. This process starts after reaping the cocoa fruits, during which the mucilage containing the cocoa beans is fermented in heaps or boxes. After drying, the dried cocoa beans are roasted, husked, and ground. Processing exerts a strong effect on the aroma profile and chemistry of cocoa beans. However, chemistry changes mainly occur during the fermentation and drying processes [13,14,15,16,17]. Usually, fermented cocoa beans are depleted of bioactive compounds but contain high amounts of key odorant volatiles [10]. Therefore, genetic factors, environmental conditions, the production life cycle and postharvest practices alter the chemical fingerprint of cocoa beans and may provide unique characteristics to cocoa products.

The complex chemical composition of cocoa is responsible for its heterogeneous health benefits. Cocoa has been recognized as an important source of polyphenols, including special flavonoid monomers (catechin and epicatechin) and oligomeric polymers (procyanidins). Polyphenols have antioxidant potential and protective roles associated with reducing the likelihood of acute myocardial infarction, death from cardiovascular disease, flow-mediated vessel dilatation, stroke and diabetes. They have anti-adiposity, anti-inflammatory, antiplatelet, anti-skin aging and fat-reducing activities, and reduce markers of insulin resistance [13,18,19,20,21,22,23]. The polyphenols in cocoa beans have also long been related to the flavor and color of chocolate [24]. The alkaloids theobromine (3,7-dimethylxanthine) and caffeine (1,3,7-trimethylxanthine) are widely present in cocoa, and a variety of pharmacological properties have been attributed to these compounds. These compounds stimulate the cardiac and respiratory system and are known for their psychoactive effects on the central nervous system by functioning as adenosine receptor antagonists and nonselective phosphodiesterase (PDE) inhibitors; and they increase metabolism [25,26,27]. In the central nervous system, theobromine is related to effects on blood pressure via peripheral physiological changes, and caffeine may exert greater CNS-mediated effects on alertness [26,28]. On the other hand, methylxanthines have been shown to stimulate lipolysis and inhibit adipogenesis, properties that might contribute to obesity control [29]. All these properties of both polyphenols and methylxanthines may be used in products prepared from cocoa beans. Therefore, an understanding of the chemical composition of cacao clones growing under different conditions is essential for extraction processes in industry.

Although a large number of studies have examined the contents of polyphenols and methylxanthines in cocoa beans from plants with different genotypes [24,30,31] or grown in different areas [5,9,10] (these references correspond only to a sample of the existing reports), to date, no study has analyzed the bioactive components by measuring a combination of these parameters. Additionally, few studies have shown the relationship between aging cocoa plants and the yield of bean production [11,32]. However, the effect of aging on the production of those secondary metabolites during the cocoa life cycle must be studied. Thus, the aim of this study was to jointly analyze the flavanol, procyanidin and methylxanthine contents in ten clones by comparing introduced clones (CCN51, EET96, ICS1, ICS60, ICS-95 and TSH565) and regional clones (FSV-41, FEAR-5, FEC-2 and TAME-2) cultivated in two different regions of Antioquia, Colombia, over two consecutive years of harvest to understand how these conditions affect the chemistry of unfermented cacao beans, and to establish the suitability of parameters related to the contents of bioactive compounds for the selection of clones for the establishment of new crops. These new crops should provide a functional market for polyphenols and methylxanthines.

## 2. Results and Discussion

Cocoa plays a relevant economic role in Colombia. The cultivation of cocoa beans has been promoted as an alternative to illegal crop production and for the reconstruction of the communities affected by armed conflict [33]. In addition, one of the activities contemplated in the cocoa farming system in Colombia has been the introduction of regional cacao clones, which are expected to be of better quality, more resistant to pathogens and exhibit higher productivity. Therefore, some clones, such as FSV-41, FEAR-5, FEC-2 and TAME-2, have been introduced that are part of a plan of gradual replacement of the universal clones. The local clone FEC-2, for example, is resistant to aggressive strains of *Moniliophthora roreri*, whereas CCN-51 is cataloged as moderately resistant [34]. However, despite the importance of this agricultural chain, currently, no chemical comparisons of introduced and regional clones from different growth areas and different harvesting years are available. Therefore, the chemical study of bioactive compounds present in each clone growing under these different conditions is important.

### 2.1. Chemical Compositions of Different Cocoa Clones

**Figure 1:**
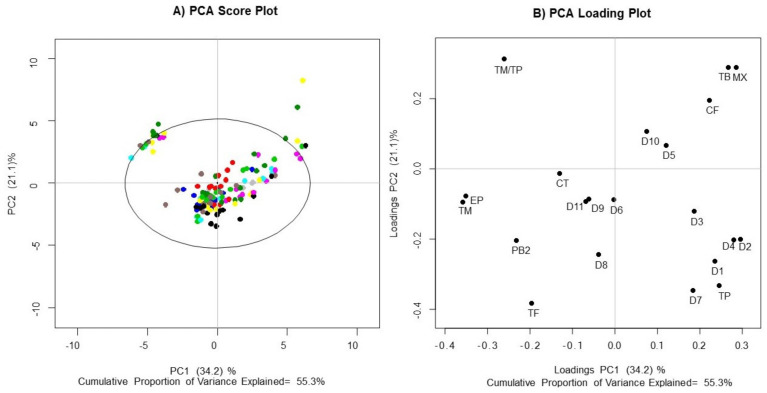
PCA plots of flavanol monomers, flavanol oligomers and methylxanthine alkaloids in *Theobroma cacao* seeds. (**A**) Score plot and (**B**) loading plot. The color code is as follows: ICS-1, yellow; EET-96, dark blue; FEC-2, light green; TAME-2, dark green, FSV-41, red; CCN-51, black; ICS-95, fuchsia; TSH-565, aqua; FEAR-5, dark gray; and ICS-60, light gray. TM: total monomers; TP: total procyanidins; MX: total methylxanthines; TM/TP: monomer/procyanidin ratio; CT: catechin; EP: epicatechin; PB2: procyanidin B2; CF: caffeine; TB: theobromine; D1–11: unknown procyanidins 1–11; TF: total flavonoids.

An analysis of the chemical compositions of cocoa clones was performed using a targeted quantitative metabolomics approach with three groups of biological compounds of interest: flavanol monomers (epicatechin and catechin), flavanol oligomers (procyanidins) and methylxanthine alkaloids (caffeine and theobromine). As has been reported by various authors, unfermented cocoa beans contain more of these metabolites than fermented samples. Therefore, the use of nonfermented cocoa beans as analytical samples was decisive [35,36]. Principal component analysis (PCA) was used to summarize the information contained in the data matrix from the HPLC-DAD-FLD analysis with dimensions of 141 × 20 (n × p). The compounds quantified in the HPLC-DAD-FLD metabolic profile corresponded to the quantitative variables (p) included in the study: catechin (CT), epicatechin (EP), procyanidin B2 (PB2), procyanidins (D1 to D11), theobromine (TB), caffeine (CF), total monomers (TM), total procyanidins (TP), monomer/procyanidin ratio (TM/TP), total methylxanthines (MX) and total flavanols (TF) (Supplementary Materials, Figure S1; Table S1). The PCA graph (Figure 1) shows that 55.3% out of total variance was explained by the first two principal components. PC1 and PC2 accounted for 34.2% and 21.1%, respectively. The variance explained for the first two principal components does not seem to be very high; however, our main interest in the PCA results was whether the separation trend between clones would be obvious. Hence, these inevitable disadvantages notwithstanding, it did not prevent us from further comparison among clones, harvest times and places of origin. The PCA map explained by three and four principal component is shown in Supplementary Materials (Figure S2). No significant differences were observed between universal and regional clones in the PCA score graph (Figure 1A). Although some cocoa samples had a higher content of monomers than of procyanidins (quadrant II), differences are not related to the clones. In addition, substantial variability was observed within the same groups of clones, since they were distributed in all quadrants (large statistical distance). Notably, the EET-96 (dark blue) and FSV-41 (red) clones displayed the least variability, since they presented the smallest statistical distance according to the score plot (Figure 1A). Both clones contained high concentrations of monomers, polymers and methylxanthines, which can be seen because they are located toward the center of the plane.

The loading plot allowed us to identify some potential associations between metabolites and each sample. The *x*-axis and *y*-axis values in the loading plot represent the contribution of each variable to the model fitting. The variables located far from the zero-point contributed more to the model, as shown in Figure 1B. On the one hand MX, TB and CF made major contributions to PC1 and PC2, resulting in a greater dispersion of the samples towards quadrant I. On the other hand, TM, EP and TM/TP, contributed to the differentiation of some samples in a defined cluster.

**Figure 2:**
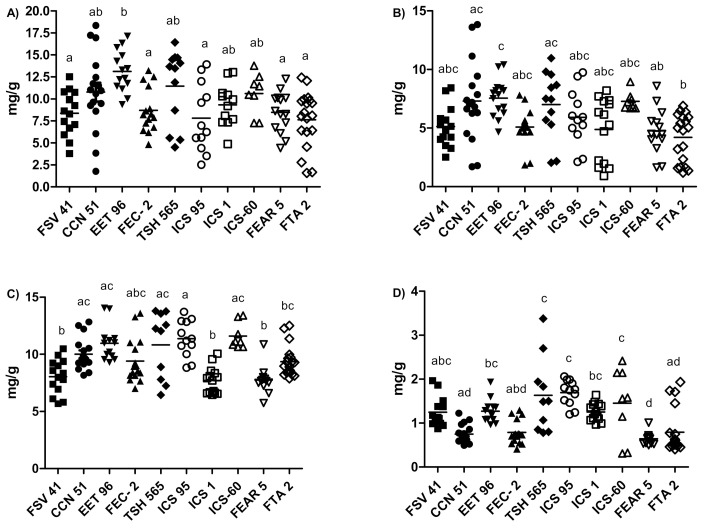
Flavanol, procyanidin and methylxanthine contents in *Theobroma cacao*. (**A**) Total monomer (catechin and epicatechin), (**B**) total procyanidin, (**C**) theobromine and (**D**) caffeine contents. Analysis was performed using one-way ANOVA. The same letters indicate non-significant differences between clones (Bonferroni’s multiple comparison test, *p* > 0.05). The analysis included the two harvest times.

The univariate analysis allowed us to establish with greater precision the contents of flavanol monomers, flavanol oligomers and methylxanthine alkaloids in each clone, and the differences between clones. According to the results shown in Figure 2, EET-96 was the only clone that did not show high dispersion among samples in polyphenol content. Likewise, this clone had the highest contents of both monomers (13.12 ± 2.30 mg/g) and procyanidins (7.56 ± 4.59 mg/g) (Figure 2A,B, respectively), followed by TSH-565 (monomers 11.46 ± 4.24 mg/g and procyanidins 7.00 ± 2.92 mg/g) and CCN-51 (monomers 10.78 ± 4.42 mg/g and procyanidins 7.31 ± 3.10 mg/g). Regarding the regional clones (FSV-41, FEC-2, TAME-2 and FEAR-5), no statistically significant differences in the monomer and procyanidin contents were observed. The monomer concentration was 8.30 ± 2.6 mg/g, and the procyanidin concentration averaged 4.79 ± 1.80 mg/g. The metabolite levels of the compounds of interest showed more promise in universal clones, especially in EET-96.

The caffeine and theobromine contents were slightly lower than the concentrations of the catechin monomers. In addition, theobromine was the predominant methylxanthine in the cocoa samples. Total methylxanthine concentrations were more homogeneous among the clones compared with polyphenol concentrations (Figure 2). Clone TSH-565 showed the greatest dispersion. Regarding the theobromine content (Figure 2C), FSV-41, ICS-1 and FEAR-5 clones, with an average level of 7.89 mg/g, had the lowest contents; and EET-96, TSH-565, ICS-95 and ICS-60 had 3.31 mg/g higher content on average. A similar trend was observed for the caffeine content (Figure 2D). The highest average content among the clones reached 1.70 mg/g. Previously, a relationship between the cocoa genotype and methylxanthine concentration was observed [5]. The theobromine–caffeine relationship facilitated the classification of clones by their origins into Criollo, Forastero and Trinitario cocoa plants. The vast majority of Colombian strains are of Trinitario origin [5].

### 2.2. Chemical Compositions of Cocoa Clones Grown in Different Areas

**Figure 3:**
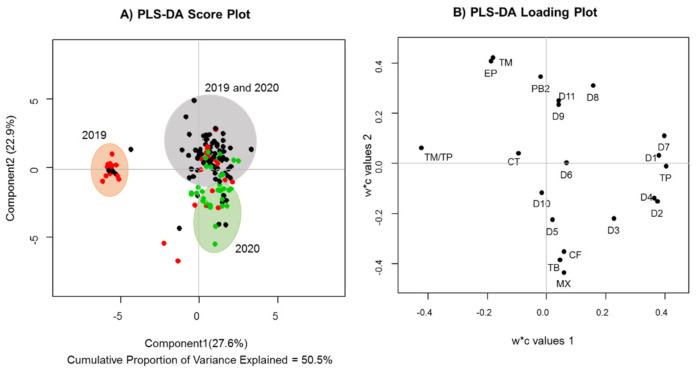
Effects of the growth areas and year of harvest on levels of the metabolites flavanol monomers, flavanol oligomers and methylxanthine alkaloids in *Theobroma cacao* seeds. Discriminant analysis PLS-DA score (**A**) and loading (**B**) plot. The color code is as follows: Chigorodó: black; Turbo: red; and Northeast region: green. TM: total monomers; TP: total procyanidins; MX: total methylxanthines; TM/TP: monomer/procyanidin ratio; CT: catechin; EP: epicatechin; PB2: procyanidin B2; CF: caffeine; TB: theobromine; D1–11: unknown procyanidins 1–11.

To better analyze the metabolic differences between groups, discriminant supervised analysis was performed to observe the effects of growing area on the levels of the main phytochemical markers we selected (Figure 3). The PLS-DA score plot shows relevant distinctions among cocoa samples of different origins. The red, black and green points represent the samples from the Turbo, Chigorodó and Northeast regions, respectively. The cocoa samples from Chigorodó and Turbo (Urabá region) are mainly distributed in the upper right and upper left quadrants, respectively. The Northeast samples are distributed along the lower and upper halves of the quadrant of the PLS-DA score plot, showing high dispersion.

The Turbo samples are characterized by higher levels of flavanol monomers than the other regions. Chigorodó samples showed high monomer and procyanidin contents. Clones from the Northeast region had higher methylxanthine content. These results are consistent with published data, where a significant effect of the cocoa-producing region on the content of the polyphenol compounds is noted [5]. Therefore, in addition to the clone of the cocoa beans, the geographic origin and harvest conditions of each region may contribute to major discrepancies observed in the results [5]. The Chigorodó and Turbo regions are characterized by a warm, humid climate with rain throughout the year. The annual rainfall is between 2000 and 2500 mm. It has an average temperature of above 24 °C. In the Northeast region of Antioquia, summers are short, hot and sultry; the winters are short and oppressive; and it is wet and cloudy year-round. During the course of the year, the temperature generally ranges from 19 to 27 °C.

The synthesis of secondary metabolites in plants is susceptible to environmental factors, meaning that these factors affect quality. Although the genotype and postharvest processing appear to affect the polyphenol composition to a greater extent than environmental conditions, the results suggest that the geographic location also affected the chemical properties of cocoa samples. In general, products generated from plant species are affected by the amounts of secondary metabolites present in plants. They ultimately affect the effectiveness, safety and reliability of the finished product [37]. Therefore, the identification of bioactive markers can contribute to the planning of interventions to increase the yields of metabolites in natural products—for example, an improvement in agronomic management. The compositions of flavanols and methylxanthines in cocoa beans obtained from the same region may be different (as evidenced in the Chigorodó samples). In addition, information on the chemical composition is important, in order to identify general and common difficulties related to the quality of a product from a specific region. In this sense, knowledge of the heterogeneity of the metabolites of interest in each clone is important for the selection of the right clone. The EET-96 clone, due to its low variability and higher contents of polyphenols and methylxanthines, is the most promising one for commercial purposes.

### 2.3. Chemical Compositions of Cocoa Clones Harvested in Different Years

The cocoa plant has different stages in its life cycle, including an initial period without yield, followed by two periods of increasing yields, the first at an increasing rate and the second at a decreasing rate, and finally a period of a decreased yield [11,38]. Although several studies have shown the relationship between the aging of cocoa plants and the yield of beans [11,32], the effect of aging on the contents of flavanol monomers, flavanol oligomers and methylxanthine alkaloids during the cocoa plant’s life cycle must be studied. In the present study, marked differences were observed in the Turbo region according to the harvest years (Figure 3). The amount of monomers produced was higher in the first year of harvest compared to the amount of procyanidins (quadrant I). No difference in the production of one type of metabolite over another was observed in the second year of harvest. In the Northeast region, a trend toward higher production of methylxanthines was observed in the second year of harvest. The levels of both types of metabolites (flavonoids and methylxanthines) were very similar in Chigorodó in both years. These results show a clear difference in metabolism according to the agronomic management in each region, which suggests the use of more standardized practices in the regions with fewer differences according to the sampling times investigated.

**Figure 4:**
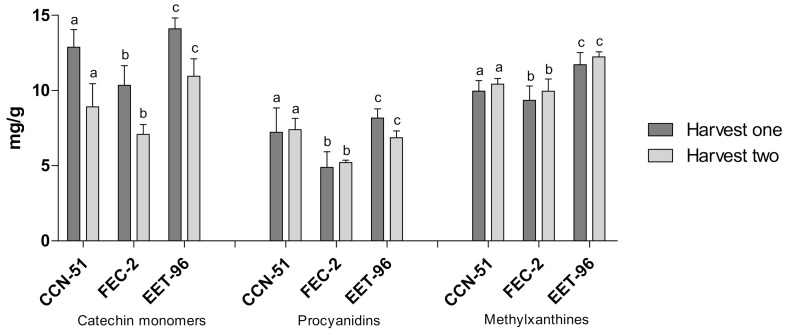
Chemical compositions of representative cocoa clones in different years of harvest (harvest one: 2019, harvest two: 2020). Analysis was performed using one-way ANOVA. The same letters indicate a non-significant difference between harvest periods for each clone (Bonferroni’s multiple comparison test, *p* > 0.05).

In the EET-96 clone, which had the highest contents of the metabolites of interest; CCN-51, as a universal clone of reference; and FEC-2, as a representative clone of the regional clones, which had the least dispersion of data, the metabolite levels were studied at the two sampling times (first and second year) to analyze changes over time (Figure 4). The amount of catechin monomers tended to decrease from one year to another in the three clones; however, the difference was not statistically significant. The opposite trend was observed for the production of methylxanthines, which tended to increase in the second year. Regarding the procyanidin oligomers, generalized behavior was not observed; however, the differences in the amounts produced were not significant between the years of observation. Based on these results, we inferred stable metabolite levels over time, regardless of the type of clone studied. However, longer studies that include the different phases of the cocoa life cycle in relation to the production of flavanol monomers, flavanol oligomers and methylxanthine alkaloids are needed.

## 3. Materials and Methods

### 3.1. Chemicals and Reagents

The solvents used were HPLC or analytical grade (Merck Chemicals, Darmstadt, Germany). The standard compounds (−)-epicatechin, (+)-catechin, caffeine and theobromine were obtained from Sigma–Aldrich (St. Louis, MO, USA). Deionized water was prepared with a Milli-Q water purification kit (Millipore, Bedford, MA, USA).

### 3.2. Samples

Seventy-one samples of T. cacao were collected in two harvesting years (November 2019 and November 2020) in farms located in Urabá (Chigorodó and Turbo) and the Northeast region of Antioquia-Colombia. At least 16 kg of ten different clones of fresh and healthy cocoa pods were collected, including six universal clones (CCN-51, EET-96, ICS-95, ICS-60, ICS-1 and TSH-565) and four regional clones (FSV-41, TAME-2, FEAR-5 and FEC-2). The ten clones were collected from at least four cocoa-producing farms in each region. In each farm, five trees per clone were sampled, from which three to four pods were collected. The pods were processed two days after harvest, for this, cocoa beans were separated from husk, and immediately, unfermented cocoa beans were freeze-dried (BK-FD12P, Biobase, Shandong, China). Dried samples were mechanically triturated to reduce the particle size (IKA*^®^* A 11 basic, IKA Works, Inc., Wilmington, NC, USA). Additionally, all powdered samples were stored in sealed plastic containers at room temperature and protected from moisture and light until further analysis using HPLC.

### 3.3. Sample Preparation

Coco samples were processed through two ultrasound-assisted extraction steps using a methodology that has been previously described and validated [39]. Approximately 100 mg of each samples was extracted with 2 mL of hexane using an ultrasonic bath (Elma P60H, Singer, Germany) at a fixed power of 700 W for 8 min and centrifuged for 15 min at 13,000 rpm. The degreased samples were vacuum-dried, and then an additional extraction process was conducted with 1.5 mL of 70% ethanol for 50 min using the same ultrasound system at 30 ± 5 °C. Then, the obtained extracts were centrifuged at 13,000 rpm for 20 min at 4 °C. The supernatant was transferred to a 2.0 mL volumetric flask. The pellet was washed with an additional volume of extraction solution (500 µL). Subsequently, the samples were again centrifuged, decanted and transferred to the same volumetric flask, where the volume was adjusted. Finally, the extracts were refrigerated at −20 °C in the dark until analysis.

### 3.4. Determination of the Polyphenol and Methylxanthine Contents

In the quantification of epicatechin, catechin, procyanidin, caffeine and theobromine levels, each extracted sample was analyzed using a previously validated method with some changes [39]. An analytical HPLC Agilent 1200 Series LC system (Agilent Technologies, Palo Alto, CA, USA) equipped with an autosampler, a diode array detector (DAD) set to 280 nm, a fluorescent detector, a quaternary pump and a vacuum degasser was used. The compounds were separated on a Zorbax column (SB-C18 RRTT, 50 × 4.6 mm × 1.8 µm) at 47.5 °C. The mobile system was composed of 0.1% acetic acid (solvent A) and acetonitrile (solvent B) with the following gradient profile: 0–1 min, 95% A; 2–11 min, 85% A; 12–15 min, 75% A; and 16–18.5 min, 95% A. The volume injected into the equipment was 3 µL with a flow rate of 1.2 mL/min. The different compounds were detected using the FLD set to a 230 nm excitation wavelength and a 320 nm emission wavelength for the analysis of flavanol monomers and procyanidins, and a DAD set to 280 nm for methylxanthines. The flavanol (catechin and epicatechin) and methylxanthine (caffeine and theobromine) compounds were identified by comparing the chromatographic data obtained with those of the standards. The assignment of the flavanol oligomer signals was performed tentatively based on our validated method [39]. An external standard method was used to quantify the compounds epicatechin, catechin, caffeine and theobromine. A relative quantification of procyanidins was performed in terms of milligram equivalents of epicatechin per gram of sample. All analyzes were performed three times for statistical purposes.

### 3.5. Statistical Analysis

One-way analysis of variance with least significant difference and Bonferroni’s multiple comparison tests was used. A *p*-value < 0.05 denoted statistical significance. The multivariate statistical analysis was performed using chemometric methods. Peaks were processed according to the retention time, and the content was reported in mg/g of sample for all compounds of interest. Chemometric modeling included partial least squares-discriminant analysis (PLS-DA) and principal component analysis (PCA). Both were applied in the statistical analysis using the MUMA package in R software (Version 3.3.1, R Core Team, Vienna, Austria). The rest of statistical study was performed using Graph Pad Prism^®^ version 8.0.2 for Windows software (Graph Pad Software, Inc., San Diego, CA, USA, 2007).

## 4. Conclusions

In general, genotype is the main factor that determines the chemistry of the cocoa beans, although significant differences were not observed between universal and regional clones in the PCA analysis. The univariate analysis allowed us to establish that EET-96 has the highest contents of both flavanol monomers and procyanidins. In addition to the clone, the geographic origin, the growing conditions of each region and the year of harvest may contribute to major discrepancies between results. Turbo cocoa samples should be noted for their higher monomer content, Chigorodó cocoa samples for the presence of both types of polyphenols (monomers and polymers) and Northeast cocoa samples for the highest methylxanthine content. The establishment of characteristics other than agronomic ones or production yields, such as the contents of compounds with beneficial properties for health, is an important milestone for decision making based on the projections of cocoa cultivation. The uses of the fruit not only in the production of fine chocolates (flavor and aroma) but also in other types of products, such as functional foods, may be developed, which would diversify the economy, generate added value, and penetrate new markets for this agricultural product for which demand is currently growing around the world.

## Data Availability

Not applicable.

## Supplementary Materials

The following supporting information can be downloaded at: https://www.mdpi.com/article/10.3390/molecules27072068/s1, Figure S1: HPLC-DAD-FLD metabolic profile of quantified compounds in Theobroma cacao L. unfermented beans, Figure S2: PCA plots with PC3 and PC4 principal components of flavanol monomers, flavanol oligomers and methylxanthine alkaloids in *Theobroma cacao* seeds. (A) Score plot and (B) loading plot. The color code is as follows: ICS-1, yellow; EET-96, dark blue; FEC-2, light green; TAME-2, dark green, FSV-41, red; CCN-51, black; ICS-95, fuchsia; TSH-565, aqua; FEAR-5, dark gray; and ICS-60, light gray. TM: total monomers; TP: total procyanidins; MX: total methylxanthines; TM/TP: monomer/procyanidin ratio; CT: catechin; EP: epicatechin; PB2: procyanidin B2; CF: caffeine; TB: theobromine; D1–11: unknown procyanidins 1–11; TF: total flavonoids.
