# Supplementary material for: Chemometric Classification of Colombian Cacao Crops: Effects of Different Genotypes and Origins in Different Years of Harvest on Levels of Flavonoid and Methylxanthine Metabolites in Raw Cacao Beans
Source: Molecules. 2022 Mar 23;27(7):2068. doi: 10.3390/molecules27072068 (PMC9000445; doi:10.3390/molecules27072068)
Supplement: Supplementary file 1 [file molecules-27-02068-s001.zip › molecules-1624893-supplementary.pdf]

molecules

MDPI

Chemometric Classification of Colombian Cacao Crops: Effects of Different Genotypes and Origins in Different Years of Harvest on Levels of Flavonoid and Methylxanthine Metabolites in Raw Cacao Beans

Catalina Agudelo [1], Susana Acevedo [1], Luis Carrillo-Hormaza [1,2], Elkin Galeano [1] and Edison Osorio [1,*]

[1] Grupo de Investigación en Sustancias Bioactivas, Facultad de Ciencias Farmacéuticas y Alimentarias, Universidad de Antioquia, Calle 70 No. 52-21, 0500100 Medellín, Colombia; catalina.agudelor@udea.edu.co (C.A.); susana.munoza@udea.edu.co (S.A.); lcarrillo@bioingred.co (L.C.-H.); elkin.galeano@udea.edu.co (E.G.)

[2] Bioingred, Spin-Off Universidad de Antioquia, 055412 Itagüí, Colombia

* Correspondence: edison.osorio@udea.edu.co; Tel./Fax: +574-219-6590

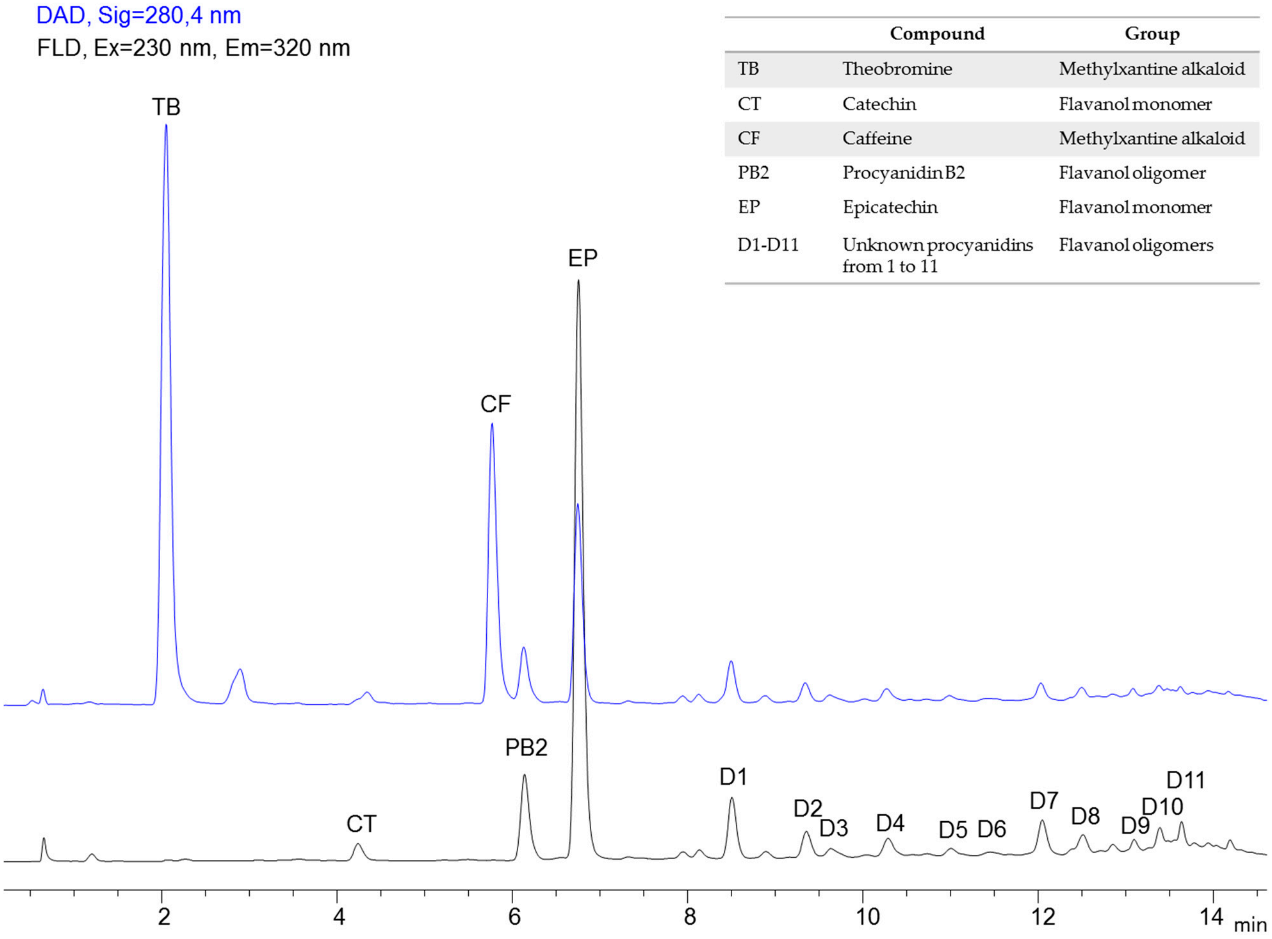

	Compound	Group
TB	Theobromine	Methylxantine alkaloid
CT	Catechin	Flavanol monomer
CF	Caffeine	Methylxantine alkaloid
PB2	Procyanidin B2	Flavanol oligomer
EP	Epicatechin	Flavanol monomer
D1-D11	Unknown procyanidins from 1 to 11	Flavanol oligomers

Figure S1. HPLC-DAD-FLD metabolic profile of quantified compounds in *Theobroma cacao* L. unfermented beans.

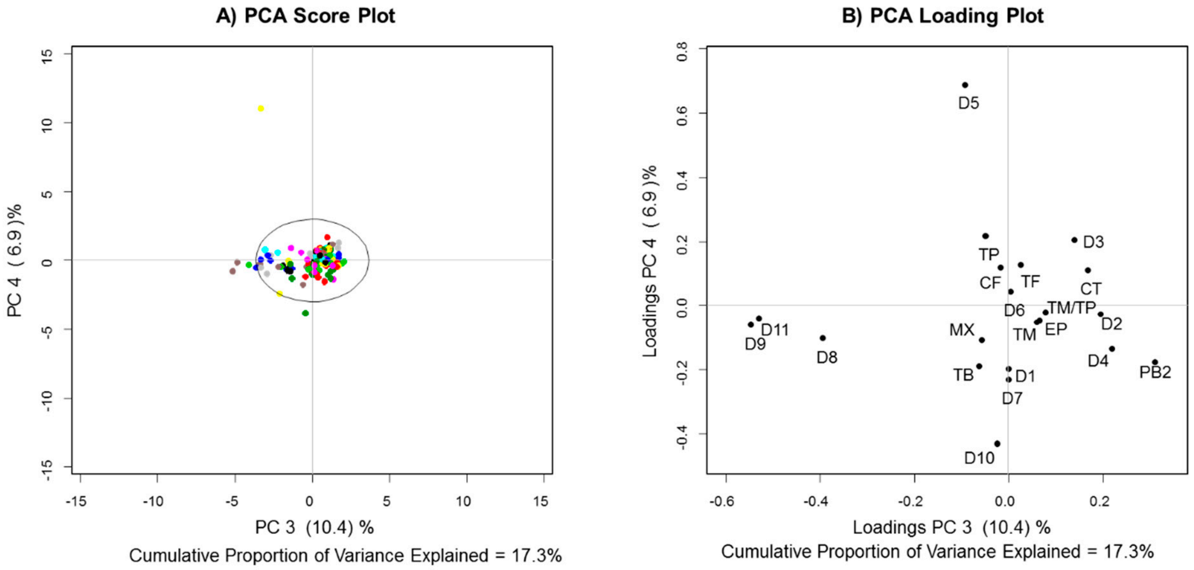

Figure S2. PCA plots with PC3 and PC4 principal components of flavanol monomers, flavanol oligomers and methylxanthine alkaloids in *Theobroma cacao* seeds. **A**) Score plot and **B**) loading plot. The color code is as follows: ICS-1, yellow; EET-96, dark blue; FEC-2, light green; TAME-2, dark green, FSV-41, red; CCN-51, black; ICS-95, fuchsia; TSH-565, aqua; FEAR-5, dark gray; and ICS-60, light gray. TM: total monomers; TP: total procyanidins; MX: total methylxanthines; TM/TP: monomer/procyanidin ratio; CT: catechin; EP: epicatechin; PB2: procyanidin B2; CF: caffeine; TB: theobromine; D1-11: unknown procyanidins 1-11; TF: total flavonoids.

The contribution ratios were 10.4% and 6.9% for PC3 and PC4, respectively. As seen from Figure S2A, the short distances between samples in the score plot indicates that there is no difference between the cocoa clones regarding the metabolites studied. FSV-41 and ISC-95 clones presented the least variability. Samples corresponded to FEAR-5, EET-96, ICS-60 and TSH-565 clones showed greater dispersion affected by the presence of metabolites D8, D9 and D11 (procyanidins) while TAME-2 clone showed dispersion affected by the content of methylxantines (MX, TB) and D10 as shown in Figure S2B, loading plot of PCA that revealed the specific components that lead to the differences between samples.

Table S1. Samples and concentrations of individual markers.

Sample	TB	CF	CT	PB2	EP	D1	D2	D3	D4	D5	D6	D7	D8	D9	D10	D11	TM	TP	TM/TP	TF	MX
	mg/g																				
CCN 10	12.21	1.02	0.37	3.47	17.97	2.70	1.78	0.87	1.39	0.76	0.57	1.44	0.63	0.00	0.00	0.00	18.34	13.61	1.35	31.95	13.23
CCN 13	12.84	0.63	0.35	3.38	16.89	2.71	1.81	0.90	1.43	0.80	0.62	1.51	0.67	0.00	0.00	0.00	17.24	13.83	1.25	31.07	12.84
EET 8	13.99	1.29	0.50	2.88	16.65	2.10	1.30	0.63	1.01	0.58	0.00	1.18	0.55	0.00	0.00	0.00	17.15	10.23	1.68	27.38	15.28
CCN 3	12.52	0.86	0.46	3.10	16.50	2.32	1.46	0.72	1.09	0.64	0.00	1.29	0.53	0.00	0.00	0.00	16.96	11.15	1.52	28.11	12.52
TSH 7	13.76	2.70	0.39	2.98	16.03	2.28	1.48	0.73	1.05	0.63	0.00	1.30	0.52	0.00	0.00	0.00	16.42	10.97	1.50	27.39	16.46
EET 11	14.04	1.37	0.44	2.91	15.90	2.15	1.37	0.69	0.95	0.61	0.00	1.20	0.53	0.00	0.00	0.00	16.34	10.41	1.57	26.75	15.41
EET 2	9.57	1.50	0.45	2.50	15.40	1.79	0.99	0.54	0.77	0.00	0.00	1.06	0.54	0.00	0.00	0.00	15.85	8.19	1.94	24.04	11.07
EET 4	9.55	0.97	0.38	2.79	14.54	2.01	1.28	0.00	0.99	0.00	0.00	1.13	0.00	0.00	0.00	0.00	14.92	8.20	1.82	23.12	9.55
TSH 4	12.05	1.83	0.37	2.09	14.35	1.30	0.64	0.38	0.54	0.35	0.28	1.11	0.82	0.58	0.00	0.58	14.72	8.67	1.70	23.39	13.88
TSH 3	7.80	1.07	0.23	2.59	14.45	1.82	1.06	0.52	0.80	0.00	0.00	1.06	0.61	0.00	0.00	0.00	14.68	8.46	1.74	23.14	8.86
TSH 6	13.80	3.38	0.30	2.33	14.17	1.66	0.91	0.49	0.75	0.42	0.00	1.12	0.70	0.55	0.00	0.64	14.47	9.57	1.51	24.04	17.18
EET 5	9.55	1.24	0.39	2.72	13.89	1.97	1.25	0.00	0.98	0.00	0.00	1.09	0.00	0.00	0.00	0.00	14.28	8.01	1.78	22.29	10.79
TSH 8	12.24	1.49	0.29	2.36	13.78	1.67	0.95	0.52	0.73	0.44	0.34	1.15	0.59	0.52	0.00	0.53	14.07	9.80	1.44	23.87	13.73
ICS95 2	11.20	1.99	0.34	2.36	13.59	0.00	0.00	0.00	0.00	0.00	0.00	0.00	0.00	0.00	0.00	0.00	13.93	2.36	5.90	16.29	13.19
CCN 5	9.49	0.76	0.00	2.45	13.79	1.66	0.89	0.00	0.70	0.00	0.00	1.04	0.64	0.00	0.00	0.00	13.79	7.38	1.87	21.17	9.49
ICS-60 1	11.09	1.56	0.36	2.50	13.41	0.00	1.28	0.72	1.00	0.59	0.00	1.13	0.00	0.00	0.00	0.00	13.77	7.22	1.91	20.99	12.65
TSH 1	8.90	0.85	0.31	2.04	13.36	0.00	0.00	0.00	0.00	0.00	0.00	0.00	0.00	0.00	0.00	0.00	13.67	2.04	6.70	15.71	8.90
TSH 2	6.44	0.78	0.30	2.17	13.17	0.00	0.00	0.00	0.00	0.00	0.00	0.00	0.00	0.00	0.00	0.00	13.47	2.17	6.21	15.64	6.44
EET 9	10.02	1.08	0.34	2.50	13.12	1.86	1.15	0.58	0.89	0.00	0.00	1.01	0.48	0.00	0.00	0.00	13.46	8.47	1.59	21.93	11.10
ICS95 1	10.83	1.47	0.30	2.12	13.02	0.00	0.00	0.00	0.00	0.00	0.00	0.00	0.00	0.00	0.00	0.00	13.32	2.12	6.28	15.44	12.29
EET 10	10.20	0.99	0.29	2.57	13.01	1.98	1.24	0.62	0.94	0.00	0.00	1.04	0.00	0.00	0.00	0.00	13.30	8.39	1.59	21.69	10.20
FEC 3	8.16	0.74	0.37	2.61	12.86	1.92	1.26	0.00	0.96	0.00	0.00	1.06	0.00	0.00	0.00	0.00	13.23	7.81	1.69	21.04	8.16
ICS1 7	8.58	1.43	0.21	2.63	12.83	2.03	1.35	0.00	1.04	0.00	0.00	1.15	0.00	0.00	0.00	0.00	13.04	8.20	1.59	21.24	10.00
ICS1 2	8.10	1.44	0.00	1.96	12.91	0.00	0.00	0.00	0.00	0.00	0.00	0.00	0.00	0.00	0.00	0.00	12.91	1.96	6.59	14.87	9.53
ICS 60 3	11.12	1.58	0.26	2.10	12.27	1.43	0.74	0.40	0.54	0.00	0.00	0.81	0.65	0.49	0.00	0.52	12.53	7.68	1.63	20.21	12.70
FEC 1	7.76	0.76	0.32	1.99	12.20	0.00	0.00	0.00	0.00	0.00	0.00	0.00	0.00	0.00	0.00	0.00	12.52	1.99	6.29	14.51	7.76
FSV 2	10.48	1.37	0.24	1.92	12.28	1.19	0.59	0.00	0.46	0.00	0.00	0.89	0.65	0.00	0.00	0.00	12.52	5.70	2.20	18.22	11.85

CCN 11	10.32	0.67	0.25	2.48	12.21	1.91	1.24	0.66	0.96	0.58	0.00	1.12	0.48	0.00	0.00	0.00	12.46	9.43	1.32	21.89	10.32
FTA 7	12.51	1.94	0.27	2.25	12.15	0.00	1.07	0.58	0.88	0.56	0.00	1.14	0.00	0.00	0.00	0.00	12.42	6.48	1.92	18.90	14.45
EET 12	10.99	1.37	0.28	2.34	12.06	1.73	1.01	0.53	0.76	0.00	0.00	1.04	0.46	0.00	0.00	0.00	12.34	7.87	1.57	20.21	12.36
FEAR 11	10.87	0.72	0.26	2.14	12.01	1.49	0.85	0.43	0.64	0.00	0.00	1.03	0.70	0.66	0.00	0.65	12.27	8.59	1.43	20.86	10.87
EET 1	9.88	1.09	0.31	1.72	11.91	0.99	0.50	0.00	0.00	0.00	0.00	0.82	0.55	0.51	0.00	0.54	12.22	5.63	2.17	17.85	10.97
FEC 4	7.00	0.53	0.35	2.38	11.87	1.85	1.23	0.00	0.95	0.00	0.00	1.06	0.00	0.00	0.00	0.00	12.22	7.47	1.64	19.69	7.00
FTA 6	12.27	1.74	0.30	2.14	11.76	0.00	1.00	0.55	0.82	0.53	0.00	1.13	0.00	0.00	0.00	0.00	12.06	6.17	1.95	18.23	14.01
ICS95 4	12.90	1.90	0.38	2.30	11.63	1.78	1.09	0.58	0.87	0.54	0.40	1.09	0.68	0.41	0.00	0.00	12.01	9.74	1.23	21.75	14.81
TSH 5	7.25	0.80	0.20	2.26	11.68	1.63	0.98	0.50	0.74	0.00	0.00	0.88	0.52	0.00	0.00	0.00	11.88	7.51	1.58	19.39	7.25
EET 6	11.41	1.60	0.28	1.88	11.50	1.46	0.64	0.00	0.55	0.00	0.00	1.01	0.62	0.50	0.00	0.00	11.78	6.66	1.77	18.44	13.01
CCN 9	10.54	0.64	0.24	2.27	11.50	1.80	1.08	0.56	0.85	0.47	0.00	1.06	0.53	0.00	0.00	0.00	11.74	8.62	1.36	20.36	10.54
ICS 60 4	13.39	0.31	0.30	2.00	11.40	1.43	0.80	0.00	0.64	0.00	0.00	0.93	0.66	0.50	0.00	0.50	11.70	7.46	1.57	19.16	13.39
CCN 6	8.17	0.71	0.00	2.04	11.51	1.37	0.76	0.00	0.60	0.00	0.00	0.92	0.62	0.00	0.00	0.00	11.51	6.31	1.82	17.82	8.17
FEC 2	8.54	0.41	0.33	1.86	11.15	0.00	0.00	0.00	0.00	0.00	0.00	0.00	0.00	0.00	0.00	0.00	11.48	1.86	6.17	13.34	8.54
ICS60 2	10.85	1.15	0.28	2.24	11.17	0.00	1.24	0.71	1.01	0.59	0.00	1.00	0.00	0.00	0.00	0.00	11.45	6.79	1.69	18.24	12.00
EET 7	11.22	1.36	0.25	1.89	11.20	1.28	0.70	0.00	0.53	0.00	0.00	0.94	0.55	0.45	0.00	0.45	11.45	6.79	1.69	18.24	12.58
FEAR 6	8.39	0.65	0.24	2.04	10.93	1.45	0.84	0.45	0.64	0.00	0.00	0.91	0.00	0.00	0.00	0.00	11.17	6.33	1.76	17.50	8.39
EET 3	9.30	1.08	0.28	1.62	10.87	0.98	0.60	0.00	0.49	0.00	0.00	0.78	0.54	0.50	0.00	0.51	11.15	6.02	1.85	17.17	10.38
FSV 1	9.38	1.87	0.96	1.92	10.18	1.62	1.04	0.53	0.83	0.54	0.41	0.96	0.60	0.00	0.00	0.00	11.14	8.45	1.32	19.59	11.25
CCN 2	8.39	0.63	0.30	1.80	10.74	0.00	0.00	0.00	0.00	0.00	0.00	0.00	0.00	0.00	0.00	0.00	11.04	1.80	6.13	12.84	8.39
CCN 7	9.29	0.59	0.00	2.02	10.94	1.43	0.80	0.00	0.63	0.00	0.00	0.88	0.57	0.00	0.00	0.48	10.94	6.81	1.61	17.75	9.29
FSV 9	7.99	1.38	0.32	2.14	10.48	1.61	1.07	0.00	0.84	0.00	0.00	0.94	0.00	0.00	0.00	0.00	10.80	6.60	1.64	17.40	9.37
FSV 14	9.94	1.97	0.26	1.99	10.49	1.65	1.08	0.55	0.85	0.55	0.00	0.99	0.52	0.00	0.00	0.00	10.75	8.18	1.31	18.93	11.91
ICS1 8	7.99	1.15	0.20	2.26	10.46	1.81	1.23	0.00	0.96	0.00	0.00	1.03	0.00	0.00	0.00	0.00	10.66	7.29	1.46	17.95	9.14
CCN 1	9.54	0.50	0.28	1.70	10.31	0.00	0.00	0.00	0.00	0.00	0.00	0.00	0.00	0.00	0.00	0.00	10.59	1.70	6.23	12.29	9.54
ICS1 1	6.55	1.12	0.00	1.54	10.48	0.00	0.00	0.00	0.00	0.00	0.00	0.00	0.00	0.00	0.00	0.00	10.48	1.54	6.81	12.02	7.66
ICS 60 6	13.28	2.42	0.00	2.29	10.47	1.87	1.21	0.65	0.93	0.54	0.00	0.97	0.49	0.00	0.00	0.00	10.47	8.95	1.17	19.42	15.70
ICS 60 5	11.75	0.32	0.25	1.80	10.14	1.26	0.70	0.00	0.56	0.00	0.00	0.82	0.57	0.52	0.00	0.52	10.39	6.75	1.54	17.14	11.75
ICS1 6	7.92	0.97	0.21	1.86	10.09	1.33	0.77	0.41	0.59	0.00	0.00	0.87	0.53	0.00	0.00	0.00	10.30	6.36	1.62	16.66	7.92
ICS 95 13	13.10	1.80	0.25	2.39	9.94	1.96	1.25	0.66	0.99	0.57	0.00	1.07	0.53	0.00	0.00	0.00	10.19	9.42	1.08	19.61	14.90
FTA 3	8.91	0.75	0.25	1.63	9.90	0.00	0.00	0.00	0.00	0.00	0.00	0.00	0.00	0.00	0.00	0.00	10.15	1.63	6.23	11.78	8.91

FEAR 2	7.87	0.53	0.25	1.72	9.88	0.00	0.00	0.00	0.00	0.00	0.00	0.00	0.00	0.00	0.00	0.00	10.13	1.72	5.89	11.85	7.87
FEAR 1	6.61	0.50	0.30	1.65	9.81	0.00	0.00	0.00	0.00	0.00	0.00	0.00	0.00	0.00	0.00	0.00	10.11	1.65	6.13	11.76	6.61
FEAR 3	8.01	0.68	0.00	1.37	10.04	0.79	0.00	0.00	0.00	0.00	0.00	0.72	0.62	0.50	0.00	0.49	10.04	4.49	2.24	14.53	8.01
EET 14	11.42	1.94	0.23	1.83	9.79	1.36	0.81	0.42	0.66	0.00	0.00	0.91	0.51	0.48	0.00	0.54	10.02	7.52	1.33	17.54	13.36
FTA 9	9.64	0.45	0.26	1.71	9.70	1.14	0.64	0.00	0.52	0.00	0.00	0.79	0.60	0.00	0.00	0.00	9.96	5.40	1.84	15.36	9.64
ICS1 4	6.68	1.07	0.00	1.70	9.95	0.00	0.00	0.00	0.00	0.00	0.00	0.00	0.00	0.53	0.00	0.00	9.95	2.23	4.46	12.18	7.75
FSV 3	8.58	0.99	0.24	1.63	9.69	1.21	0.69	0.00	0.47	0.00	0.00	0.83	0.45	0.00	0.00	0.00	9.93	5.28	1.88	15.21	8.58
FEAR 4	5.74	0.54	0.00	1.32	9.88	0.73	0.00	0.00	0.00	0.00	0.00	0.77	0.61	0.51	0.00	0.49	9.88	4.43	2.23	14.31	5.74
FTA 10	8.58	0.41	0.25	1.85	9.54	1.38	0.87	0.00	0.66	0.00	0.00	0.79	0.00	0.00	0.00	0.00	9.79	5.55	1.76	15.34	8.58
FTA 4	8.24	0.52	0.26	1.59	9.48	0.00	0.00	0.00	0.00	0.00	0.00	0.00	0.00	0.00	0.00	0.00	9.74	1.59	6.13	11.33	8.24
CCN 8	8.69	0.52	0.00	1.81	9.62	1.29	0.72	0.00	0.58	0.00	0.00	0.78	0.57	0.00	0.00	0.51	9.62	6.26	1.54	15.88	8.69
ICS95 5	12.84	1.75	0.32	1.47	9.25	1.45	0.91	0.51	0.73	0.48	0.38	0.93	0.61	0.39	0.00	0.00	9.57	7.86	1.22	17.43	14.59
FTA 2	8.43	0.46	0.33	1.37	9.19	0.00	0.00	0.00	0.00	0.00	0.00	0.00	0.00	0.00	0.00	0.00	9.52	1.37	6.95	10.89	8.43
FTA 8	8.51	0.61	0.23	1.73	9.26	1.24	0.74	0.00	0.57	0.00	0.00	0.78	0.00	0.00	0.00	0.00	9.49	5.06	1.88	14.55	8.51
CCN 12	9.39	0.97	0.00	1.82	9.47	1.38	0.75	0.43	0.63	0.00	0.71	0.91	0.56	0.00	0.00	0.00	9.47	7.19	1.32	16.66	9.39
EET 13	11.02	1.18	0.25	1.78	9.13	1.34	0.76	0.00	0.60	0.00	0.00	0.90	0.50	0.41	0.00	0.00	9.38	6.29	1.49	15.67	12.20
CCN 17	9.82	0.78	0.00	1.93	1.93	1.93	0.82	0.45	0.67	0.45	0.00	0.96	0.61	0.47	0.00	0.00	9.26	7.86	1.18	17.13	9.82
FSV 4	9.15	1.18	0.24	1.61	9.01	1.11	0.63	0.00	0.49	0.00	0.00	0.77	0.53	0.00	0.00	0.00	9.25	5.14	1.80	14.39	10.33
ICS-1 5	7.71	0.99	0.20	1.51	9.00	1.04	0.57	0.00	0.46	0.00	0.00	0.81	0.53	0.00	0.00	0.36	9.20	5.28	1.74	14.48	7.71
FEC 8	10.45	0.71	0.28	1.72	8.62	1.37	0.80	0.44	0.65	0.00	0.00	0.86	0.47	0.00	0.00	0.00	8.90	6.31	1.41	15.21	10.45
TSH 9	13.56	1.50	0.24	1.76	8.47	1.46	1.00	0.57	0.81	0.53	0.00	0.90	0.43	0.00	0.00	0.00	8.71	7.46	1.17	16.17	15.06
FEC 15	8.15	0.61	0.18	1.51	8.48	1.04	0.60	0.35	0.47	0.00	0.00	0.75	0.40	0.00	0.00	0.00	8.66	5.12	1.69	13.78	8.15
FSV 13	6.94	1.11	0.32	1.76	8.33	1.33	0.90	0.00	0.72	0.00	0.32	0.79	0.00	0.00	0.00	0.00	8.65	5.82	1.49	14.47	8.05
FEAR 9	7.77	0.65	0.00	1.67	8.63	1.23	0.75	0.00	0.56	0.00	0.00	0.79	0.47	0.00	0.00	0.00	8.63	5.47	1.58	14.10	7.77
CCN 14	9.15	0.63	0.00	1.79	8.60	1.41	0.82	0.44	0.66	0.00	0.00	0.90	0.59	0.45	0.00	0.00	8.60	7.06	1.22	15.66	9.15
FTA 1	8.40	0.48	0.33	1.19	8.12	0.00	0.00	0.00	0.00	0.00	0.00	0.00	0.00	0.00	0.00	0.00	8.45	1.19	7.10	9.64	8.40
FSV 7	7.80	1.00	0.00	1.59	8.30	1.17	0.69	0.00	0.52	0.00	0.00	0.81	0.00	0.00	0.00	0.00	8.30	4.78	1.74	13.08	7.80
FEAR 13	9.57	1.50	0.22	1.78	8.07	1.55	1.09	0.57	0.86	0.56	0.00	0.89	0.00	0.00	0.00	0.00	8.29	7.30	1.14	15.59	11.07
FEC 14	7.66	0.53	0.20	1.47	8.08	0.95	0.54	0.00	0.43	0.00	0.00	0.78	0.48	0.00	0.00	0.00	8.28	4.65	1.78	12.93	7.66
FEAR 7	7.67	0.66	0.22	1.53	8.03	1.13	0.61	0.00	0.49	0.00	0.00	0.80	0.53	0.00	0.00	0.00	8.25	5.09	1.62	13.34	7.67
FEC 6	8.93	0.74	0.00	1.41	8.21	0.91	0.47	0.00	0.45	0.00	0.00	0.82	0.58	0.44	0.00	0.46	8.21	5.54	1.48	13.75	8.93
FTA 11	8.22	0.48	0.22	1.47	7.88	1.02	0.60	0.00	0.48	0.00	0.00	0.71	0.51	0.00	0.00	0.00	8.10	4.79	1.69	12.89	8.22
ICS-1 3	6.63	1.21	0.00	1.62	8.09	0.00	0.00	0.00	0.00	0.00	0.00	0.00	0.00	0.00	0.00	0.00	8.09	1.62	4.99	9.71	7.84

FTA 13	9.98	0.57	0.24	1.58	7.78	1.20	0.76	0.40	0.58	0.00	0.00	0.83	0.59	0.44	0.00	0.00	8.02	6.38	1.26	14.40	9.98
FEC 2	9.24	0.70	0.22	1.58	7.67	1.17	0.68	0.00	0.55	0.00	0.00	0.76	0.46	0.00	0.00	0.00	7.89	5.20	1.52	13.09	9.24
FTA 15	9.74	0.78	0.23	1.66	7.54	1.39	0.89	0.47	0.70	0.45	0.00	0.86	0.47	0.00	0.00	0.00	7.77	6.89	1.13	14.66	9.74
FEC 7	8.39	0.75	0.00	1.43	7.70	1.08	0.63	0.00	0.48	0.00	0.00	0.72	0.51	0.43	0.00	0.00	7.70	5.28	1.46	12.98	8.39
ICS-1 12	10.05	1.64	0.00	1.70	7.70	1.54	1.12	0.60	0.94	0.56	0.00	0.91	0.00	0.00	0.00	0.00	7.70	7.37	1.04	15.07	11.69
FSV 6	9.24	1.51	0.00	1.38	7.44	1.06	0.63	0.00	0.46	0.00	0.00	0.70	0.00	0.00	0.00	0.00	7.44	4.23	1.76	11.67	10.75
ICS-1 9	6.60	1.11	0.00	1.58	7.36	1.32	0.90	0.49	0.69	0.43	0.00	0.75	0.00	0.00	0.00	0.00	7.36	6.16	1.19	13.52	7.70
ICS-1 11	9.57	1.48	0.00	1.62	7.35	1.46	1.07	0.58	0.89	0.54	0.00	0.88	0.00	0.00	0.00	0.00	7.35	7.04	1.04	14.39	11.04
FEAR 8	7.51	0.68	0.00	1.36	7.31	0.91	0.50	0.00	0.43	0.00	0.00	0.78	0.53	0.00	0.00	0.00	7.31	4.51	1.62	11.82	7.51
ICS 60 7	10.65	2.14	0.00	1.49	7.26	1.31	0.90	0.51	0.75	0.49	0.00	0.80	0.45	0.00	0.00	0.00	7.26	6.70	1.08	13.96	12.79
ICS 60 8	10.65	2.14	0.00	1.49	7.26	1.31	0.90	0.51	0.75	0.49	0.00	0.80	0.45	0.00	0.00	0.00	7.26	6.70	1.08	13.96	12.79
FEC 5	8.41	0.55	0.00	1.37	7.24	0.99	0.60	0.00	0.48	0.00	0.00	0.77	0.47	0.00	0.00	0.00	7.24	4.68	1.55	11.92	8.41
FSV 5	8.36	1.11	0.17	1.23	6.92	0.87	0.49	0.00	0.37	0.00	0.00	0.67	0.40	0.00	0.00	0.00	7.09	4.03	1.76	11.12	9.47
ICS-95 6	8.84	1.24	0.18	1.71	6.80	1.49	1.08	0.00	0.89	0.00	0.00	0.89	0.00	0.00	0.00	0.00	6.98	6.06	1.15	13.04	10.08
FEC 9	8.99	0.76	0.00	1.39	6.78	1.12	0.71	0.00	0.59	0.00	0.00	0.74	0.44	0.00	0.00	0.00	6.78	4.99	1.36	11.77	8.99
FEAR 5	7.53	1.01	0.00	1.11	6.75	0.74	0.00	0.00	0.00	0.00	0.00	0.74	0.53	0.16	0.00	0.00	6.75	3.28	2.06	10.03	8.54
FSV 10	7.38	1.13	0.00	1.26	6.70	0.93	0.53	0.00	0.41	0.00	0.00	0.70	0.43	0.00	0.00	0.00	6.70	4.26	1.57	10.96	8.51
FEC 10	11.07	1.20	0.20	1.56	6.23	0.00	1.10	0.00	0.95	0.61	0.00	0.86	0.00	0.00	0.00	0.00	6.43	5.08	1.27	11.51	12.27
FTA16	9.34	0.63	0.00	1.49	6.43	1.27	0.80	0.43	0.66	0.00	0.00	0.78	0.44	0.00	0.00	0.00	6.43	5.87	1.10	12.30	9.34
FTA 17	11.37	1.71	0.21	1.40	6.22	1.24	0.85	0.47	0.73	0.48	0.00	0.76	0.00	0.00	0.00	0.00	6.43	5.93	1.08	12.36	13.08
FTA 14	8.97	0.53	0.00	1.40	6.25	1.15	0.74	0.42	0.59	0.00	0.00	0.72	0.00	0.00	0.00	0.00	6.25	5.02	1.25	11.27	8.97
ICS-95 8	11.37	1.77	0.00	1.62	6.24	1.54	1.10	0.58	0.89	0.55	0.00	0.79	0.00	0.00	0.00	0.00	6.24	7.07	0.88	13.31	13.13
FEC 11	11.01	1.30	0.20	1.53	6.00	0.00	1.10	0.00	0.95	0.62	0.00	0.86	0.00	0.00	0.00	0.00	6.20	5.06	1.23	11.26	12.30
FEAR 10	7.25	0.54	0.00	1.12	6.11	0.87	0.54	0.00	0.39	0.00	0.00	0.69	0.48	0.00	0.00	0.00	6.11	4.09	1.49	10.20	7.25
CCN 15	9.24	1.23	0.00	1.52	6.05	1.36	0.89	0.51	0.72	0.42	0.00	0.79	0.42	0.00	0.00	0.00	6.05	6.63	0.91	12.68	10.46
FSV 8	5.82	0.87	0.00	1.15	5.92	0.84	0.49	0.00	0.00	0.00	0.43	0.61	0.00	0.00	0.00	0.00	5.92	3.52	1.68	9.44	5.82
EET 15	9.57	1.50	0.24	1.24	5.53	1.05	0.73	0.42	0.61	0.00	0.00	0.62	0.00	0.00	0.00	0.00	5.77	4.67	1.24	10.44	11.07
ICS-95 3	11.87	1.66	0.00	1.28	5.63	1.13	0.77	0.00	0.63	0.00	0.00	0.65	0.00	0.00	0.00	0.00	5.63	4.46	1.26	10.09	13.53
TSH 11	12.57	1.94	0.00	1.45	5.58	1.35	0.96	0.53	0.80	0.51	0.00	0.77	0.00	0.00	0.00	0.00	5.58	6.37	0.88	11.95	14.51
ICS-95 7	9.01	1.20	0.15	1.53	5.43	1.43	1.07	0.00	0.81	0.00	0.00	0.86	0.00	0.00	0.00	0.00	5.58	5.70	0.98	11.28	10.21
TSH 10	9.57	1.50	0.00	1.30	5.36	1.18	0.86	0.48	0.69	0.47	0.00	0.71	0.00	0.00	0.00	0.00	5.36	5.69	0.94	11.05	11.07
FEAR 12	7.67	0.54	0.00	1.18	5.20	1.00	0.68	0.00	0.54	0.00	0.00	0.63	0.00	0.00	0.00	0.00	5.20	4.03	1.29	9.23	7.67
FSV 11	6.10	0.95	0.00	1.08	5.01	0.79	0.50	0.00	0.39	0.00	0.00	0.51	0.00	0.00	0.00	0.00	5.01	3.27	1.53	8.28	6.10

ICS-1 13	8.35	1.36	0.00	1.19	4.87	1.10	0.75	0.42	0.61	0.00	0.00	0.65	0.00	0.00	0.00	0.00	4.87	4.72	1.03	9.59	9.71
FEC 12	13.59	1.22	0.00	1.28	4.83	1.20	0.83	0.46	0.71	0.44	0.00	0.72	0.00	0.00	0.00	0.00	4.83	5.64	0.86	10.47	14.81
FTA 12	9.29	1.45	0.00	0.98	4.56	0.85	0.56	0.00	0.48	0.00	0.00	0.56	0.00	0.00	0.00	0.00	4.56	3.43	1.33	7.99	10.75
TSH 12	9.57	1.50	0.00	1.14	4.51	1.08	0.82	0.46	0.67	0.47	0.00	0.66	0.00	0.00	0.00	0.00	4.51	5.30	0.85	9.81	11.07
FEAR 14	9.57	1.50	0.00	1.18	4.42	1.17	0.88	0.49	0.70	0.49	0.00	0.68	0.00	0.00	0.00	0.00	4.42	5.59	0.79	10.01	11.07
ICS-95 9	10.02	2.06	0.00	1.21	4.41	1.15	0.77	0.43	0.61	0.00	0.00	0.67	0.41	0.00	0.00	0.00	4.41	5.25	0.84	9.66	12.08
CCN 16	9.65	0.62	0.00	1.07	3.85	1.04	0.76	0.42	0.64	0.00	0.00	0.60	0.00	0.00	0.00	0.00	3.85	4.53	0.85	8.38	9.65
FSV 12	5.69	1.03	0.00	0.87	3.80	0.71	0.46	0.00	0.00	0.00	0.00	0.48	0.00	0.00	0.00	0.00	3.80	2.52	1.51	6.32	6.71
ICS-95 10	13.72	1.99	0.00	1.16	3.52	1.31	0.96	0.51	0.83	0.51	0.00	0.66	0.00	0.00	0.00	0.00	3.52	5.94	0.59	9.46	15.70
FTA 5	7.89	0.51	0.39	0.78	2.41	0.82	0.60	0.00	0.51	0.00	0.00	0.49	0.00	0.00	0.00	0.00	2.80	3.20	0.88	6.00	7.89
ICS-95 11	10.74	1.52	0.00	0.95	2.53	1.09	0.81	0.47	0.68	0.44	0.00	0.56	0.00	0.00	0.00	0.00	2.53	5.00	0.51	7.53	12.26
FEC 13	13.27	1.10	0.00	0.82	2.34	0.91	0.75	0.45	0.70	0.48	0.00	0.57	0.00	0.00	0.00	0.00	2.34	4.68	0.50	7.02	14.37
CCN 4	10.81	1.08	0.36	0.61	1.42	0.77	0.68	0.43	0.65	0.42	0.00	0.50	0.00	0.00	0.00	0.00	1.78	4.06	0.44	5.84	11.89
FTA 18	9.33	0.51	0.00	0.00	1.68	0.64	0.49	0.00	0.45	0.00	0.00	0.00	0.00	0.00	0.00	0.00	1.68	1.58	1.06	3.26	9.33
FTA 19	8.12	0.51	0.00	0.65	1.59	0.75	0.00	0.00	0.51	0.00	0.00	0.43	0.00	0.00	0.00	0.00	1.59	2.34	0.68	3.93	8.12
ICS-1 10	6.45	1.26	0.00	0.00	1.07	0.49	0.43	0.00	0.00	0.00	0.00	0.00	0.00	0.00	0.00	0.00	1.07	0.92	1.16	1.99	7.71
ICS-1 14	6.79	1.39	0.00	0.00	1.02	0.49	0.43	0.00	0.00	6.79	0.00	0.00	0.00	0.00	0.00	0.00	1.02	7.71	0.13	8.73	8.17

TM: total monomers**;** **TP:** total procyanidins; **MX:** total methylxanthines; **TM/TP:** monomer/procyanidin ratio; **CT:** catechin; **EP:** epicatechin; **PB2:** procyanidin B2; **CF:** caffeine; **TB:** theobromine; **D1-11:** unknown procyanidins 1-11; **TF:** total flavonoids.
